# The mAbXcite platform modifies the tumor microenvironment when applied to an immune-oncology anti-CTLA4 antibody

**DOI:** 10.1186/2051-1426-3-S2-P414

**Published:** 2015-11-04

**Authors:** Isabelle Sansal-Castellano, Mark Carlson, Gabriel Reznik, James Siedlecki, John Kane, Zuzana Dostalova, Hua Miao, Ifat Rubin-Bejerano

**Affiliations:** 1 ImmuneXcite Inc., Lexington, MA, USA

## 

The recent successes in the immunotherapy field demonstrate that a successful immune response to cancer can lead to meaningful anti-tumor responses. Antibodies blocking immune checkpoint molecules (e.g. CTLA4, and PD-1) lead to effective T cell responses especially in tumors that are immune active.

Immune cells that have not received as much attention in cancer immunotherapy are neutrophils. Neutrophils are the most abundant white blood cells, which protect us from microbial infections. They are the first line of innate defense: they arrive early on in the course of infection, phagocytose, and release the content of their granules, including reactive oxygen species and enzymes. Neutrophils also communicate with other immune cells, such as macrophages, dendritic cells, and T cells, by releasing cytokines and chemokines, leading to an orchestrated innate and adaptive immune response.

There has long been a call for a targeted recruitment of these professional killers to fight cancer. However, an approach that mediates the recruitment of neutrophils has to lead to acute as opposed to chronic inflammation to overcome the suppressive environment that tumors surround themselves with.

We have developed a novel immunotherapy platform technology, termed mAbXcite, which directs and activates neutrophils to initiate a robust innate and adaptive immune response to kill cancer cells in a targeted manner. The targeting is achieved by using monoclonal antibodies that are chemically linked to beta-1, 6-glucan, a saccharide found on the cell walls of fungal species which recruits and activates neutrophils. The resulting mAb construct attracts mediators of acute inflammation, notably neutrophils, which are followed by macrophages and T cells.

We have shown that this platform leads to stasis and regressions in tumor models that are resistant to monoclonal antibodies that target peripheral cancer antigens, including validated antibodies (e.g. trastuzumab and cetuximab) and experimental mouse antibodies.

Here, we will show that the platform can be applied to antibodies that target the tumor microenvironment resulting in greater efficacy and changes in infiltrating immune cells.

